# Spinal Muscular Atrophy Carrier Screening: Assessment of Provider Knowledge and Clinical Practice

**DOI:** 10.1002/pd.70023

**Published:** 2025-11-16

**Authors:** Melissa Riegel, Whitney Bender, Elizabeth Critchlow, Lorraine Dugoff

**Affiliations:** ^1^ Department of Obstetrics and Gynecology Perelman School of Medicine Division of Maternal Fetal Medicine University of Pennsylvania Philadelphia Pennsylvania USA; ^2^ Department of Obstetrics and Gynecology Sidney Kimmel Medical College Division of Maternal Fetal Medicine Thomas Jefferson University Philadelphia Pennsylvania USA

## Abstract

**Objective:**

The American College of Obstetricians and Gynecologists (ACOG) recommends offering spinal muscular atrophy (SMA) carrier screening (CS) preconception or prenatally. This study aimed to determine provider knowledge of SMA and SMA CS practice patterns and to describe the relationship between knowledge and comfort while discussing screening and results.

**Method:**

Prenatal providers completed an anonymous web‐based survey on SMA knowledge, CS practice patterns, and comfort in interpretation of results. Data were summarized with descriptive statistics. The relationship between provider training and SMA knowledge with provider comfort was analyzed.

**Results:**

75% (112/150) of providers responded and 64.6% completed the survey. Participants varied in roles and years of experience. The mean score on knowledge was 3.8/8 (47.5%) with 20.6% of respondents scoring ≥ 75% and 51.6% scoring ≥ 50%. Knowledge did not vary with years of experience. Although 91.3% of providers offer SMA screening, less than 25% reported complete comfort discussing screening and results. Comfort correlated with role and experience. Providers who felt completely comfortable discussing SMA screening had higher knowledge scores.

**Conclusion:**

Although the majority of providers offer SMA CS, provider knowledge regarding SMA is low, and most are not comfortable discussing screening and results.

## Summary

- What is already known about this topic?
  ◦ Previous studies have shown poor provider comprehension of genetic screening
  ◦ No studies specifically looking at spinal muscular atrophy (SMA) carrier screening (CS) knowledge and comfort
- What does this study add?
  ◦ The majority of prenatal care providers offer SMA CS
  ◦ Provider knowledge regarding SMA is low
  ◦ Most providers are not completely comfortable discussing SMA screening and test results
  ◦ This study, demonstrating poor SMA knowledge and comfort, shows a critical need for improved provider education and support.

## 1 Introduction

Spinal muscular atrophy (SMA) is the most common neurodegenerative genetic disorder, occurring in approximately 1 in 6000 to 1 in 10,000 live births. SMA is associated with insufficient levels of the survival motor neuron (SMN) protein which causes the progressive degeneration of motor neurons in the brainstem and spinal cord, leading to atrophy of skeletal and respiratory muscles, progressive weakness, and, when untreated, the most severe forms can result in early death due to respiratory failure [1]. There have been promising recent advancements in the treatment of SMA including gene therapy and orphan drug therapies which have demonstrated improved outcomes in children and adults. Although research in this area is ongoing, current studies suggest improved prognosis with earlier treatment, likely related to the irreversibility of neuronal death [2]. Since SMA is the leading genetic cause of infant death and earlier initiation of treatment is associated with a better prognosis, there has been an emphasis on carrier screening in both the preconception and pregnant population. Current ACOG guidelines recommend that screening be offered for all considering pregnancy, with an emphasis on the importance of pre‐ and post‐test counseling [3]. As of 2016, approximately 20% of patients offered SMA screening at the time of genetic counseling underwent screening [4].

Carrier screening (CS) for SMA measures the number of copies of the survival motor neuron 1 (SMN1) gene. Potential results on the SMA screen include negative carrier, positive carrier, and linked‐variant results. A noncarrier is expected to have two or more SMN1 gene copies, while a carrier will have only one functional copy. SMA carriers most commonly have one functional SMN1 gene on one chromosome and an SMN1 gene deletion on the other chromosome (“trans”). Carriers can also have two functional SMN1 gene copies on one chromosome (“cis”) and none on the other chromosome (“2 + 0” genotype), or one chromosome with a non‐functional SMN1 gene with a point mutation or a microdeletion. The “2 + 0” genotype occurs in 5%–8% of the general population, with black individuals of sub‐Saharan African heritage having a higher frequency of this change. PCR‐based carrier testing is unable to reliably identify this genotype. Therefore, the sensitivity for carrier screening in the black population is 70% compared to 87%–95% in the non‐black population [5].

More recently, variants in intron 7 and exon 8 of the SMN1 gene have been found to be more prevalent in carriers of the “2 + 0” genotype. In the Ashkenazi Jewish population, these variants occur in approximately 20% of patients with the “2 + 0” genotype compared to < 1% of non‐carriers [6]. Testing for these variants has been added by some laboratories for routine CS. Importantly, the presence of the variant does not confirm the “2 + 0” genotype but rather increases the likelihood of this genotype, thereby impacting the residual risk after screening.

Partner testing is recommended for patients who are identified to be SMA carriers. Follow‐up for patients receiving a linked variant result is individualized pending patient counseling. Patient counseling and follow‐up recommendations, however, may vary based on provider comfort or knowledge of SMA screening including an understanding of the significance of a linked variant result. As of January 2025, there have been no published reports on provider understanding of SMA screening or result interpretation using a combination of search terms in PubMed including “SMA screening,” “SMA results,” “SMA carrier,” and “SMA result interpretation.” We suspected there is wide variation in provider knowledge and recommendations for follow‐up care after a positive or linked‐variant SMA CS result. Therefore, the objective of this study was to determine baseline provider knowledge of SMA and SMA CS practice patterns in a large academic OBGYN department and to describe the relationship between provider knowledge and training with comfort in discussing screening and results.

## 2 Methods

We performed a cross‐sectional survey of prenatal care providers at the University of Pennsylvania Health System from January 2022 to January 2023. The study was deemed to be exempt by the institutional review board.

Providers were invited to complete an anonymous twenty‐item survey via the Qualtrics online platform between January 2022 and January 2023. The survey was developed using the literature published by the American College of Medical Genetics (ACMG) and ACOG and with expert input from a board‐certified maternal‐fetal medicine (MFM)‐geneticist and prenatal genetic counselors [1, 3, 4, 5]. The survey was then piloted among Obstetric‐Gynecology (OBGYN) providers who were not included in the final survey population.

The survey was designed to assess providers' SMA CS knowledge, result interpretation, and practice patterns. Two questions were used to determine demographic factors, including years in practice and clinical role (i.e., MFM attending, MFM fellow, OBGYN generalist, OBGYN resident, certified nurse midwife, and other including advanced practice provider or nurse practitioner). Eight questions addressed clinical knowledge (Table 1) and ten questions addressed clinical practice (Table 2).

**TABLE 1:** Knowledge‐based SMA survey questions.

Knowledge‐based questions	Multiple choice answer options^a^
Which of the following is true regarding SMA?	*a. It leads to atrophy of skeletal muscle and overall weakness* b. It leads to intellectual disability later in life c. All types of SMA result in difficulty breathing d. The earlier the symptoms present, the milder the case will be
What is the inheritance pattern of SMA?	a. Autosomal dominant *b. Autosomal recessive* c. X‐linked recessive d. Mitochondrial
The incidence of SMA is approximately:	a. 1 in 2000 to 1 in 6000 live births *b. 1 in 6000 to 1 in 10,000 live births* c. 1 in 10,000 to 1 in 14,000 live births d. 1 in 14,000 to 1 in 18,000 live births
Which of the following is true regarding the genes responsible for SMA?	a. SMN2 is considered the active gene for survival motor neuron protein production *b. More than 98% of SMA patients have an abnormality in both SMN1 genes* c. A higher number of SMN2 copies is associated with a worse prognosis d. Two copies of a linked variant must be present in order to develop SMA
What is the carrier frequency of SMA in caucasian population?	a. 1 in 10 *b. 1 in 45* c. 1 in 100 d. 1 in 500
What is the carrier frequency of SMA in black population?	a. 1 in 10 b. 1 in 45 *c. 1 in 100* d. 1 in 500
What would be the risk of an affected child if a patient were found to be an SMA carrier and her black partner's SMA carrier status is unknown?	a. 1 in 100 *b. 1 in 400* c. Cannot be calculated
The attached picture would yield what results on SMA carrier screening (choose all that may apply). 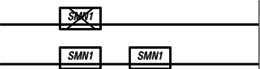	a. Normal *b. Carrier* *c. Linked variant* d. Affected

*Note:* Correct answers *italicized*.

Abbreviation: SMA: spinal muscular atrophy.

^a^ Participants able to answer “I don't know” for each question.

**TABLE 2:** Clinical practice SMA survey questions.

Clinical practice questions	Multiple choice answer options
I offer SMA screening for all patients at their first pregnancy visit:	a. Always b. > 50% of the time c. < 50% of the time d. Never
What factors influence your decision to offer SMA screening (you may select multiple answers):	a. My perception of patient values b. My perception of patients' abilities to understand the screening process c. My understanding of SMA d. Whether there is time during office visit e. I order SMA screening without discussion with patient f. Other ‐ fill in the blank:
How comfortable are you with discussing SMA screening?	a. Completely comfortable b. Somewhat comfortable c. Somewhat uncomfortable d. Not at all comfortable
Which aspects of screening are you least comfortable discussing (you may select multiple answers):	a. Genetic inheritance b. Clinical symptoms or prognosis of disease c. Interpretation of screening results d. Next steps in care after positive screening result e. My perception of patients' abilities to understand the screening process f. Other ‐ fill in the blank:
How comfortable are you with discussing SMA screening results?	a. Completely comfortable b. Somewhat comfortable c. Somewhat uncomfortable d. Not at all comfortable
Please elaborate which results you are uncomfortable discussing (you may select multiple answers):	a. Positive carrier screening b. Linked variant c. Negative carrier screening d. None ‐ I am comfortable discussing all results
What would be your next step for a patient with a linked variant? (you may select multiple answers):	a. Recommend partner testing b. Recommend genetic counseling c. Discuss carrier risk d. No further steps e. Other ‐ fill in the blank:
Are your follow‐up recommendations for patients with a linked variant the same regardless of number of SMN1 copies?	a. Yes b. No
What would be your next step in a patient with a positive carrier screening? (you may select multiple answers):	a. Recommend partner testing b. Recommend genetic counseling c. No further steps d. Other—fill in the blank:
What would be your next step in a patient with a negative carrier screening but a positive family history for SMA?	a. Recommend partner testing b. Recommend genetic counseling c. No further steps d. Other—fill in the blank:

Abbreviation: SMA: spinal muscular atrophy.

Participants were identified through listservs of the outpatient practices affiliated with the University of Pennsylvania and invitations were sent via institutional email addresses. The listserv included all prenatal providers (150), minus those who had participated in survey testing, within the OBGYN department who see patients at five different outpatient sites. All prenatal care providers are faculty, including physicians, nurse practitioners, and nurse midwives, affiliated with our academic institution or resident physicians. There are no private or non‐academic practices. Outpatient sites include an outpatient office within the hospital, outpatient offices at locations separate from the hospital, and maternal fetal medicine outpatient offices. These practices provide individual prenatal care using a shared practice model, in which patients see multiple prenatal care providers throughout their pregnancies. Prenatal education on genetic screening tests is not standardized across these practices but may include direct education from the provider during the visit, information from nursing educators, and educational material distributed on paper or electronically through patient messaging portals. Participants were excluded from the study if they did not complete the survey in its entirety.

Questions addressing knowledge were scored as correct (1) or incorrect (0), and the total knowledge score for each participant was calculated. Questions regarding clinical practice are reported as descriptive statistics. Given the exploratory nature of this survey, a passing score was not predetermined. Scores of > 50% and > 75% were utilized after survey completion to compare knowledge among providers. Provider training was considered by both the provider's professional role (i.e., advanced practice provider, current resident, current fellow, or attending physician) and years of clinical experience. The relationship of provider training and SMA knowledge with provider comfort in SMA screening and result interpretation was analyzed. Chi‐square tests were used to compare categorical data or Wilcoxon‐rank sum tests to compare continuous variables. A *p*‐value < 0.05 was considered significant.

## 3 Results

One hundred and fifty providers were invited to participate in the study. 75% (112/150) initiated the survey and 64.6% (97/150) completed the survey. Survey participants varied in roles and years of clinical experience. The majority of OBGYN providers completing the survey played the role of academic specialist or resident and 1–4 years of experience in clinical practice.

**FIGURE 1:**
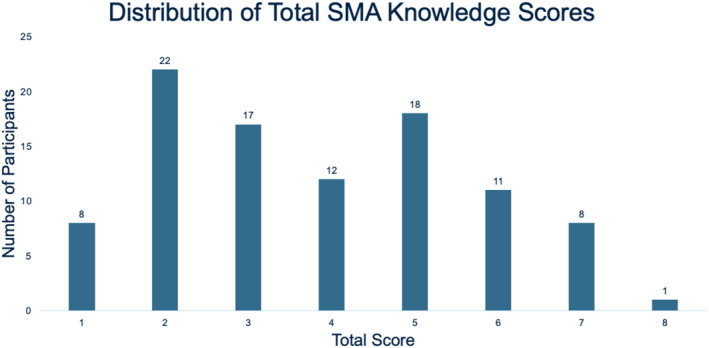
Results of knowledge portion of survey.

The mean score on the knowledge portion of the survey was 3.8 ± 1.8 out of 8 (47.5%) with 20.6% of respondents scoring ≥ 75% and 51.6% scoring ≥ 50% (Figure 1). MFM providers had the greatest mean total scores compared to non‐MFM providers, with mean scores of 5.5 ± 1.4 for MFM attendings and 4.3 ± 0.5 for MFM fellows (Table 3, *p* < 0.001). MFM providers also had significantly higher total knowledge scores ≥ 75% and total knowledge scores ≥ 50% compared to other providers. Test performance did not vary with years in practice (Table 3).

**TABLE 3:** SMA disease: screening knowledge and clinical practice.

	Provider role *N* (%)							Years of experience *N* (%)				
	MFM attending^a^ (*n* = 17)	MFM fellow^a^ (*n* = 4)	OB/Gyn Generalist^b^ (*n* = 30)	OB/Gyn Resident^b^ (*n* = 33)	CNM^c^ (*n* = 7)	Other (incl. NP)^d^ (*n* = 6)	*p*‐value	1–4 (*n* = 37)	5–10 (*n* = 25)	11–15 (*n* = 9)	16+ (*n* = 26)	*p*‐value
Clinical knowledge												
Total score^e^	5.5 (1.4)	4.3 (0.5)	3.7 (1.7)	3.3 (1.7)	1.9 (0.7)	4.2 (2.3)	< 0.001*	3.4 (1.7)	4.2 (1.7)	4.1 (2.0)	4.0 (2.1)	0.31
Total knowledge score ≥ 75%	10 (58.8)	0 (0)	4 (13.3)	4 (12.1)	0 (0)	2 (33.3)	0.001*	5 (13.5)	4 (16)	3 (33.3)	8 (30.8)	0.26
Total knowledge score ≥ 50%	16 (94.1)	4 (100)	14 (46.7)	13 (39.4)	0 (0)	3 (50)	< 0.001*	15 (40.5)	18 (72.0)	5 (55.6)	12 (46.2)	0.1
Clinical practice												
Comfort discussing screening^f^	13 (86.7)	1 (25)	21 (72.4)	15 (48.3)	4 (57.1)	6 (100)	0.003*	16 (46)	21 (87.5)	7.9 (77.8)	16 (66.7)	0.005*
Comfort discussing results^f^	12 (80)	0 (0)	22 (75.9)	10 (32.2)	5 (71.4)	6 (100)	0.001*	12 (34.2)	18 (75)	8 (88.9)	17 (70.8)	0.011*

*Note:* Data *n* (%) unless otherwise specified.

^*^ Denotes a *p*‐value < 0.05.

^a^ Maternal Fetal Medicine.

^b^ Obstetrician‐Gynecologist.

^c^ Certified Nurse Midwife.

^d^ Nurse Practitioner.

^e^ Mean (Standard Deviation).

^f^ Defined as completely or somewhat comfortable. Data available for 92 subjects.

Regarding clinical practice, 91.3% of providers offer SMA screening at the first prenatal visit, with no difference based on role or years in practice. The most common provider considerations when offering screening included offering for all patients as standard of care (35.9%), clinician understanding of screening (13.0%), and clinician values (8.7%). Only 22 (23.9%) participants reported complete comfort discussing SMA screening, while 38 (41.3%) reported being somewhat comfortable. Participants were least comfortable discussing symptoms or prognosis of SMA (19.6%), interpretation of screening results (10.9%), and genetic inheritance pattern (7.6%).

With respect to result interpretation, 19 (20.7%) reported complete comfort discussing SMA results, while 36 (39.1%) reported being somewhat comfortable. Participants reported that they were most uncomfortable discussing the linked variant result (32.6%). When faced with a linked variant result, most (32.6%) participants offered or recommended both partner testing and genetic counseling, followed by genetic counseling alone (28.3%). 58 (37%) participants agreed that follow‐up for linked variant results would change depending on the number of SMN1 copies present.

Comfort discussing screening and test results correlated with clinical role and years in practice. Participants who felt completely comfortable discussing SMA screening were more likely to score ≥ 75% or ≥ 50% on SMA knowledge (*p* < 0.005). Comfort discussing results was correlated to score ≥ 50% on SMA knowledge (*p* = 0.007).

## 4 Discussion

In this study, the majority of prenatal care providers at a large academic OBGYN department offered SMA CS to patients at the first prenatal visit. Overall provider knowledge regarding SMA is low, with MFM providers scoring the highest on the knowledge component of the survey. Most providers do not feel completely comfortable discussing SMA screening or test results with patients. Participants who scored higher on SMA knowledge were more likely to report comfort discussing SMA screening and results.

The uptake of SMA screening in the United States in 2016 was approximately 20%, and prior studies have demonstrated significant health care provider knowledge gaps as a barrier to implementing genetic screening [4]. In a survey of 632 ACOG members on their practice patterns regarding prenatal and preconception cystic fibrosis (CF) carrier screening, more than half of respondents (59.5%) reported an inability to interpret a positive screening test, and 58.9% reported a lack of familiarity with genetics and CF [7, 8]. Darcy et al. reported that 17.7% of obstetricians were unable to interpret basic cystic fibrosis (CF) carrier screening results and 43% were unaware of CF carrier rates, sensitivity of CF screening, and residual risk after a negative screen [9]. Another cross‐sectional survey of clinicians at two unaffiliated, tertiary academic institutions reported that only 36%–42% of clinicians correctly answered knowledge questions about prenatal genetic screening for fetal aneuploidy and diagnostic testing [4, 8]. The authors of this and similar studies concluded that there is a need for targeted educational efforts for providers [10, 11]. Our study included providers in an urban, tertiary academic setting. Therefore, these results may be less generalizable to providers in rural settings or in private practices. Our study is unique in that we focused on provider knowledge and genetic screening practice patterns specifically regarding SMA, which is more complex due to the variable clinical phenotypes associated with the number of SMN1 and SMN2 gene copies and the potential to receive a linked variant test result. Our findings support the need for similar educational efforts regarding SMA CS.

Many providers in the United States have introduced SMA CS into their clinical practice in response to the 2017 ACOG Committee Opinion that recommended offering SMA screening to all women who are considering pregnancy or are currently pregnant [3, 12]. However, the increase in the number of SMA tests since the publication of the ACOG Committee Opinion has been significantly lower compared to the rate of carrier screening for cystic fibrosis, which is also recommended by ACOG. It is possible that SMA carrier screening has not been well adopted due to the complexity of the screening and interpretation of the results [12]. SMA CS result management is a complicated topic to master and can be a clinical challenge for providers [12]. Our study found that providers in this tertiary urban academic center were deficient in SMA knowledge and uncomfortable counseling patients regarding SMA CS and results. The association of provider knowledge with comfort found in this study suggests that targeted educational efforts may improve the rates of provider comfort in discussing SMA CS and results. Future work should focus on the identification and development of optimal approaches to educate providers on SMA as well as the interpretation and management of carrier screening results. This endeavor should be applied to other genetic conditions as well. Additionally, further work is needed to determine the type of follow‐up pursued by patients who receive an abnormal SMA CS result, and the patient or healthcare factors that may influence these decisions.

Our study has a number of strengths. There was a 65% completion rate of the survey, which is comparable to other published survey‐based studies [13]. The survey was standardized, which allowed for minimization of biases between participants. Nonetheless, our study has several limitations. The results represent a single health system, which may limit the generalizability of our findings. There were fifteen participants who started but did not complete the survey. Reasons for these partial responses are not known; however, nonresponse may have resulted in an over‐ or underestimation of provider knowledge or misrepresentation of provider practice depending on the reason for survey non‐continuation. If a provider found the survey too difficult, for example, our results may overestimate provider knowledge. Alternatively, if a provider does not routinely discuss SMA carrier screening and did not complete the survey for that reason, then our results may not be accurately reflective of clinical practice. Finally, this survey study was susceptible to self‐selection bias, which may have led to over‐estimation of knowledge and comfort scores.

## 5 Conclusion

Although the majority of prenatal care providers in our academic health system offer SMA CS, provider knowledge regarding SMA is low, and most providers are not comfortable discussing SMA screening and test results. Educational measures should be undertaken to improve provider, and by proxy, patient understanding of available CS options and the interpretation and management of CS results.

## Funding

The authors have nothing to report.

## Ethics Statement

This study was exempted by the University of Pennsylvania Institutional Review Board on 2/17/22 (protocol #850756). Participant consent was obtained prior to participation.

## Conflicts of Interest

The authors declare no conflicts of interest.

## Data Availability

The data that support the findings of this study are available from the corresponding author upon reasonable request.
